# Heparin-Induced Thrombocytopenia in a Patient With Postoperative Pulmonary Embolism: D-dimer Re-elevation and Progressive Pulmonary Artery Thrombosis

**DOI:** 10.7759/cureus.100446

**Published:** 2025-12-30

**Authors:** Satoshi Kurisu, Hitoshi Fujiwara, Yutaka Tanaka

**Affiliations:** 1 Department of Cardiology, National Hospital Organization Hiroshima Nishi Medical Center, Otake, JPN; 2 Department of Orthopedics, National Hospital Organization Hiroshima Nishi Medical Center, Otake, JPN

**Keywords:** 4ts score, anticoagulant therapy, computed tomography pulmonary angiography, thromboembolic disease, venous thromboembolism (vte)

## Abstract

Heparin-induced thrombocytopenia (HIT) is a rare but serious immune-mediated complication of heparin therapy, characterized by thrombocytopenia and paradoxical thrombosis. We report a case of HIT in an 83-year-old woman who developed postoperative pulmonary embolism (PE). Continuous unfractionated heparin was initiated, leading to initial improvement. However, seven days after PE and heparin initiation, thrombocytopenia and D-dimer re-elevation occurred without new symptoms. Her 4Ts score was 7, prompting a switch from heparin to argatroban. HIT antibodies were subsequently confirmed, and computed tomography (CT) revealed a new thrombus in the main pulmonary artery. Argatroban was later replaced with edoxaban after platelet recovery. Both thrombocytopenia and D-dimer levels gradually improved, and the patient was discharged 30 days after PE onset. Outpatient follow-up CT confirmed complete thrombus resolution. Clinicians should maintain a high index of suspicion for HIT in heparin-treated patients presenting with thrombocytopenia, with supportive laboratory findings such as unexpected D-dimer elevation, even in the absence of new or worsening symptoms. Early recognition and prompt management are essential to improve outcomes and prevent severe complications or death.

## Introduction

Heparin-induced thrombocytopenia (HIT) is a serious, immune-mediated complication of heparin therapy, characterized by the paradoxical combination of thrombocytopenia and an increased risk of thrombosis. If unrecognized, HIT can rapidly lead to life-threatening complications [1-4].

HIT primarily affects hospitalized or recently discharged patients receiving heparin, particularly unfractionated heparin. Although its incidence is relatively low, the associated risk of arterial and venous thrombosis is substantial [5-7]. Because clinical manifestations may be subtle or nonspecific, early recognition based on clinical suspicion and appropriate diagnostic tools, such as the 4Ts score [8-10] and HIT antibody testing [10], is essential.

Here, we present a case of HIT that developed after unfractionated heparin therapy for postoperative pulmonary embolism (PE). Notably, the case was characterized by re-elevation of D-dimer levels and progressive pulmonary artery thrombosis on serial computed tomography (CT) scans, despite the absence of new or worsening symptoms, highlighting the diagnostic challenge posed by atypical presentations of HIT.

## Case presentation

An 83-year-old woman with no underlying health conditions presented with hip pain following a fall and was admitted to the hospital. Laboratory tests revealed no anemia, normal liver and kidney function, but hypoalbuminemia and an elevated D-dimer level of 76.7 µg/mL (Table 1). She was diagnosed with a left femoral neck fracture, which was surgically repaired. Postoperatively, the patient developed hypoxemia and tachycardia.

**Table 1:** Laboratory data

Variables	Initial presentation	Reference ranges
White blood cell count (/μL)	8.8 × 10^3^	3.3 - 8.6 × 10^3^
Red blood cell count (/μL)	4.36 × 10^6^	3.86 - 4.92 × 10^6^
Hemoglobin (g/dL)	13.2	11.6 - 14.8
Platelet count (/μL)	288 × 10^3^	158 - 348 × 10^3^
Aspartate aminotransferase (U/L)	14	13 - 30
Alanine aminotransferase (U/L)	13	7 - 23
Lactate dehydrogenase (U/L)	215	124 - 222
Total protein (g/dL)	5.3	6.6 - 8.1
Albumin (g/dL)	2.4	4.1 - 5.1
Blood urea nitrogen (mg/dL)	12.4	8 - 20
Creatinine (mg/dL)	0.52	0.46 - 0.79
C-reactive protein (mg/dL)	0.13	0 - 0.14
D-dimer (µg/mL)	76.7	0 - 1.1

**Figure 1:**
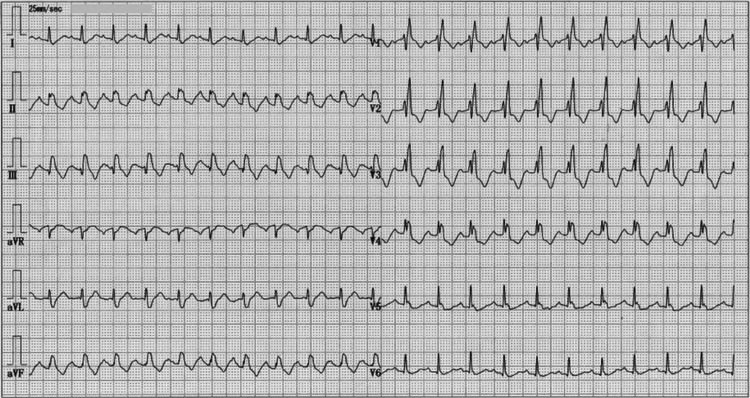
Electrocardiogram The electrocardiogram showed sinus tachycardia with right bundle branch block.

At that time, her vital signs were as follows: pulse rate, 130 beats per minute; blood pressure, 122/90 mmHg; and oxygen saturation, 90% while receiving 3 L/min of supplemental oxygen via nasal cannula. The electrocardiogram showed sinus tachycardia with right bundle branch block (Figure 1).

**Figure 2:**
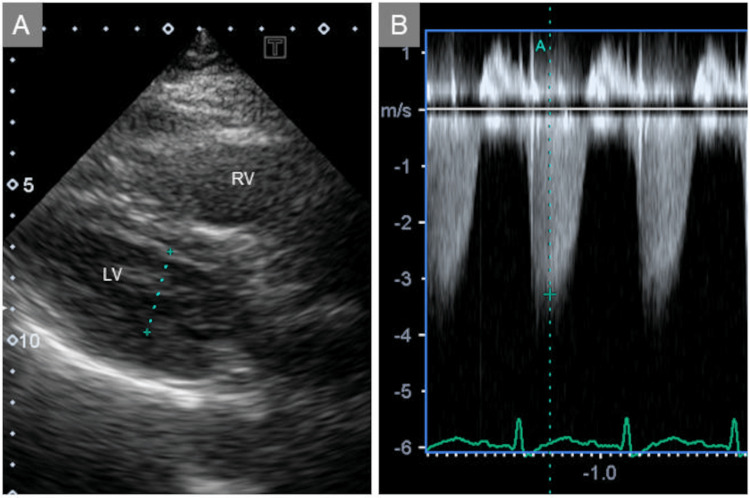
Transthoracic echocardiographic images Transthoracic echocardiography revealed right ventricular enlargement (A) and moderate tricuspid regurgitation with a peak velocity of 3.2 m/s (B). RV, right ventricle; LV, left ventricle

Transthoracic echocardiography revealed right ventricular enlargement and moderate tricuspid regurgitation, with a peak velocity of 3.2 m/s (Figure 2A and Figure 2B). These findings indicated right ventricular pressure overload.

**Figure 3:**
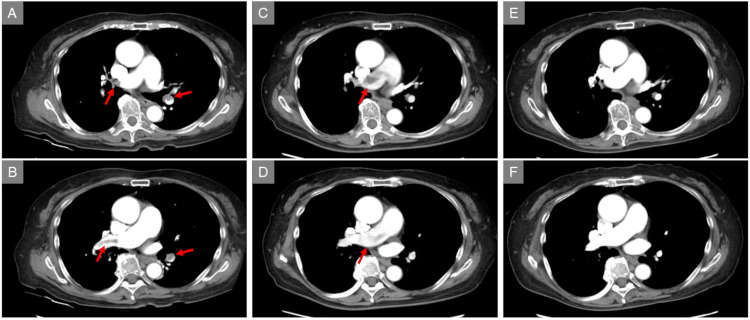
Serial CT images Contrast-enhanced CT revealed filling defects in both pulmonary arteries, consistent with thrombi (A and B, arrows). Follow-up CT revealed a filling defect consistent with newly formed thrombus in the main pulmonary artery (C and D, arrows). Outpatient follow-up CT confirmed complete resolution of the thrombus (E and F). CT, computed tomography

Contrast-enhanced CT revealed filling defects in both pulmonary arteries, consistent with thrombi (Figure 3A and Figure 3B, arrows).

**Figure 4:**
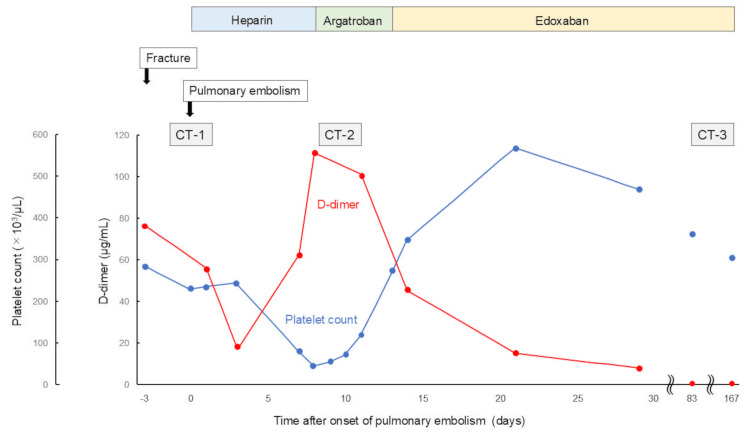
Clinical course Timeline shows changes in platelet counts and D-dimer levels, along with anticoagulation therapy and key clinical events during the perioperative period. CT, computed tomography

The diagnosis of PE was confirmed by imaging modalities. As the patient was hemodynamically stable, continuous heparin infusion was promptly initiated and titrated according to activated partial thromboplastin time (APTT), considering both its reversibility and the risk of postoperative bleeding (Figure 4). Her vital signs, oxygenation, and symptoms gradually improved over two days. Three days after PE onset and heparin initiation, the patient remained hemodynamically stable, and follow-up blood tests showed a gradual decrease in D-dimer levels.

Seven days after PE onset and heparin initiation, thrombocytopenia and D-dimer re-elevation were observed despite clinical stability, representing a critical diagnostic turning point (Figure 4). At this time, the patient’s 4Ts score was seven, strongly supporting the clinical suspicion of HIT [8-10]. Blood sampling for HIT antibody testing was performed [10]. Heparin infusion was discontinued and switched to argatroban [10-12]. The platelet count ultimately reached a nadir of 46×10³/µL. Follow-up CT revealed a filling defect consistent with newly formed thrombus in the main pulmonary artery (Figure 3C and Figure 3D, arrows). The HIT antibody test was subsequently found to be positive, leading to the diagnosis of HIT.

Thirteen days after PE onset, partial resolution of thrombocytopenia was confirmed, with a sustained increase in the platelet count and no evidence of bleeding. Based on the clinical stability and improvement in laboratory findings, anticoagulation was switched from argatroban to edoxaban, a direct oral anticoagulant. Both thrombocytopenia and D-dimer elevation gradually improved, and the patient was discharged 30 days after PE onset.

Outpatient follow-up CT confirmed complete resolution of the thrombus (Figure 3E and Figure 3F). At the six-month follow-up, the patient remained clinically stable.

## Discussion

In this report, we showed a case of HIT in a patient with postoperative PE following heparin exposure. Notably, D-dimer re-elevation was observed in parallel with progressive pulmonary artery thrombosis. The prompt switch from heparin to argatroban, followed by transition to edoxaban, led to thrombus resolution without hemodynamic compromise, emphasizing the importance of close monitoring for HIT.

Venous thromboembolism is a major concern after lower extremity surgery due to immobility and venous stasis. The cumulative incidence of PE after major orthopedic procedures is reported to be around 2%, with most cases arising from deep vein thrombosis within 72 hours postoperatively [13]. In patients with fractures, particularly those of the femoral neck, D-dimer is often elevated even in the absence of deep vein thrombosis [14]. Interpretation can be challenging, as perioperative levels may also rise due to tissue injury, inflammation, and immobilization. Although D-dimer is highly sensitive for thrombosis, its specificity is limited in this setting [15].

DOACs are increasingly used as an alternative for venous thromboembolism [16]. However, their use in the immediate postoperative setting may be limited due to bleeding risk. In this case, PE developed immediately after surgery, prompting the selection of continuous heparin infusion because of its short half-life and reversibility. Heparin dose was titrated according to APTT, allowing a balance between thrombosis and bleeding risks. The patient had no new or worsening symptoms after starting heparin. However, the combination of thrombocytopenia and D-dimer re-elevation suggested the development of HIT. Postoperative thrombocytopenia is relatively common and may result from hemodilution, surgical consumption, infection, or drug exposure. Such causes typically occur within the first few days after surgery and are not usually associated with progressive thrombosis [17]. In contrast, the delayed onset of thrombocytopenia approximately one week after heparin exposure, together with new pulmonary artery thrombosis, strongly supported the diagnosis of HIT in this case.

While thrombocytopenia is an expected finding in HIT, few reports have highlighted the behavior of D-dimer levels. Although D-dimer elevation is nonspecific in the perioperative setting, re-elevation during ongoing anticoagulation should raise concern for persistent thrombin generation. Zhang et al. described a patient who developed HIT during prophylactic nadroparin therapy and exhibited marked D-dimer elevation [18], and Yu et al. reported a similar observation in a patient with lung cancer receiving enoxaparin [19]. Taken together with our findings, these reports suggest that temporal changes in D-dimer levels may serve as a supportive clinical clue for HIT when interpreted in the appropriate clinical context. The 4Ts scoring system proved valuable, indicating a high pretest probability and prompting HIT antibody testing that established the diagnosis [8-10]. According to a meta-analysis, the absolute risk of HIT is relatively low, 2.6% with unfractionated heparin and 0.2% with low-molecular-weight heparin [2]. Nevertheless, it can result in serious complications. Early recognition, including careful monitoring of platelet counts and attention to temporal changes in D-dimer levels, is crucial to prevent abrupt clinical worsening. Finally, this is a single-case report; therefore, the observations should be interpreted cautiously and require confirmation in larger studies. In particular, the interpretation of D-dimer levels is limited, as postoperative elevation and inflammation may have confounded the assessment of thrombotic activity.

Non-heparin anticoagulants remain the standard treatment for HIT. Although there is growing evidence supporting the off-label use of DOACs in carefully selected stable patients [20], their use in the postoperative setting remains limited. In Japan, argatroban continues to be the only approved agent for HIT management, offering a short half-life, predictable anticoagulant effect, and reversibility, features that are particularly advantageous in postoperative patients and allow careful dose titration. In the present case, the use of argatroban enabled effective thrombus management while minimizing bleeding risk.

## Conclusions

In conclusion, clinicians should maintain a high index of suspicion for HIT in heparin-treated patients presenting with thrombocytopenia, with supportive laboratory findings such as unexpected D-dimer elevation, even in the absence of new or worsening symptoms. Early recognition and prompt management are essential to improve outcomes and prevent severe complications or death. Moreover, this case illustrates that HIT-related thrombosis can progress silently, with new thrombus formation occurring even in clinically stable patients, highlighting the importance of continued vigilance and careful monitoring throughout the course of heparin therapy.
